# Antibacterial activity and biocompatibility of silver coating via aerosol deposition on titanium and zirconia surfaces

**DOI:** 10.1186/s40729-023-00488-w

**Published:** 2023-09-04

**Authors:** Sunyoung Choi, Ye-Hyeon Jo, Jung-Suk Han, Hyung-In Yoon, Jae-Hyun Lee, In-Sung Luke Yeo

**Affiliations:** 1 Department of Prosthodontics, School of Dentistry and Dental Research Institute, Seoul National University, 101 Daehak-Ro, Jongro-Gu, Seoul, 03080 Korea; 2 Department of Prosthodontics, One-Stop Specialty Center, Seoul National University Dental Hospital, Seoul, Korea; 3 Dental Research Institute, Seoul National University, Seoul, Korea

**Keywords:** Silver, Titanium, Zirconia, Aerosol deposition, Bacterial proliferation, Biocompatibility

## Abstract

**Purpose:**

The purpose of this in vitro study was to investigate the antibacterial effect and biocompatibility of silver coatings via aerosol deposition on titanium and zirconia surfaces.

**Methods:**

The surfaces of titanium and zirconia specimens were polished and coated with silver via aerosol deposition. After silver coating, the elemental composition, surface roughness and amount of silver released from the coated surfaces were measured. The bacterial growth on the silver-coated surfaces was investigated via crystal violet assay after incubation with *Streptococcus gordonii* for 24 h, *Fusobacterium nucleatum* for 72 h and *Porphyromonas gingivalis* for 48 h. Human gingival fibroblasts and mouse preosteoblasts were also cultured on the silver-coated specimens to examine the biocompatibility of the coating.

**Results:**

After silver coating via aerosol deposition, the surface roughness increased significantly, and the released silver ranged from 0.067 to 0.110 ppm. The tested bacteria formed significantly less biofilm on the silver-coated titanium surfaces than on the uncoated titanium surfaces. In contrast, biofilm formation on the silver-coated zirconia surfaces was greater than that on the uncoated zirconia surfaces. Human gingival fibroblasts and mouse preosteoblasts proliferated on the silver-coated surfaces without significant differences from the uncoated surfaces.

**Conclusions:**

Silver coating via aerosol deposition provided an antibacterial effect against oral bacteria on titanium surfaces, whereas it promoted more bacterial growth on zirconia surfaces. The proliferation of fibroblasts and osteoblasts was not significantly affected by the silver coating on both titanium and zirconia surfaces.

## Background

The use of dental implants has become a widely accepted and preferred treatment modality to rehabilitate patients with edentulous sites [1–3]. With the improvement of implant materials and treatment procedures, the long-term survival rates of dental implants have been reported to be higher [1, 4]. However, despite the high survival rate of dental implants, the risk of complications remains.

Peri-implantitis is one of the representative complications of dental implants. It is a host response to the microbial challenge occurring in tissues surrounding implants and characterized by the destructive inflammation in the connective tissue and the progressive loss of supporting bone, which can lead to implant loss [5–8]. Since the normal flora lives in the oral cavity, the surfaces of implant prostheses in the oral cavity inevitably become habitats for bacteria to proliferate [9]. Furthermore, bacterial proliferation on implant surfaces is closely related to peri-implantitis. Several specific periodontal pathogens, such as *Porphyromonas gingivalis*, were observed more frequently in peri-implantitis than in healthy tissue surrounding implants [10], and a history of chronic periodontitis and poor plaque control are known risk factors for peri-implantitis with strong evidence [7, 11]. In an animal study, the progression of peri-implantitis was induced by breaks of the mucosal seal around the implants and submucosal bacterial biofilm formation [12].

To evade biofilm formation on the surfaces of dental implants, various surface treatments, such as topographical modifications or loading of antibacterial agents on surfaces, have been introduced [13, 14]. In the surface coating method, effective antibacterial agents and durable coating methods are required for reliable inhibition of biofilm formation on the implant surfaces. Silver is a widely used inorganic agent with antibacterial effects in various fields, including medical implants [15, 16]. Doping silver directly on the surfaces without additional layers, growing a titanium oxide layer with silver, and depositing a coating with silver on the surfaces were proposed as techniques for silver application on titanium implant surfaces [14]. Aerosol deposition is a method to coat surfaces with ceramic or ceramic mixtures at room temperature without a high-temperature sintering process [17]. Durable coating via aerosol deposition on the titanium surface has also been reported [18].

Titanium has long been established as the standard material for dental implants with a proven record of success [1, 4, 19]. Its physical properties and biocompatibility are suitable for use as an implant material. However, with increasing esthetic demands on dental treatments, the gray color of titanium has become a major drawback. Zirconia not only has proper physical properties and excellent biocompatibility, but also has outstanding esthetics; thus, its use as an implant material is rapidly expanding [20, 21]. Furthermore, although still controversial, some studies have shown that zirconia is more resistant to bacterial colonization than titanium and can potentially be beneficial against bacterial contamination [22–25]. However, zirconia implants are not completely free from peri-implant mucositis or peri-implantitis [26]. Both titanium and zirconia surfaces require extra treatment to reduce the potential for bacterial colonization that can cause peri-implant inflammation.

The purpose of this in vitro study was to examine the antibacterial effect of silver coating on titanium and zirconia surfaces. Silver was coated on the surfaces via aerosol deposition method and the biocompatibility of the coating was also investigated. The null hypothesis of this study was that titanium and zirconia surfaces coated with silver via aerosol deposition would have antibacterial effect and biocompatibility.

## Methods

### Specimen preparation

Titanium specimens were machined from grade 4 titanium bars (Seoul Titanium Co., LTD., Siheung, Korea) into disc shapes 20 mm in diameter and 0.5 mm in thickness. Three percent yttria-stabilized tetragonal zirconia polycrystalline discs that were 15 mm in diameter and 2 mm in thickness were prepared through cold isostatic pressing of the powder mixture (Tosoh, Tokyo, Japan) at 200 MPa, followed by sintering at 1500 °C for 2 h. The titanium and zirconia discs were polished following a previously published method [25] until the surface roughness was lower than 0.01 μm. The polished discs were coated with silver using the aerosol deposition method. First, the discs as substrates were sequentially washed using acetone, ethanol and water and mounted in the deposition chamber. Commercially available silver nanoparticles (ENB KOREA Co., LTD., Daejeon, Korea) mixed with alumina powder (Showa Denko, Tokyo, Japan) at a concentration of 1 wt% were preheated and aerosolized by vibration with a carrier gas in the aerosol chamber. The silver aerosol was accelerated by the pressure difference between the two chambers and deposited onto the substrates through a slit nozzle at room temperature. The thickness of the coating was confirmed by scanning electron microscopy to be approximately 1 μm.

### Surface characterization

The coated surfaces were observed using field emission scanning electron microscopy (SEM, S-4700, Hitachi High-Tech Corporation, Tokyo, Japan), and elemental analysis of the surfaces was performed using energy dispersive X-ray spectroscopy. The arithmetic mean heights of the surfaces of uncoated and coated specimens were measured using a three-dimensional confocal laser microscope (LSM 800, Zeiss, Jena, Germany). The amount of silver released from each coated disc was measured by incubating it in a tube containing phosphate-buffered saline (PBS) at 37 °C. After 24 h of incubation, the disc was removed from the tube, and the amount of silver in the solution was measured using inductively coupled plasma–mass spectrometry (ICP-MS, NexION 350D, Perkin-Elmer, Waltham, MA, USA).

### Bacterial adhesion and proliferation on the surface

Based on previously published data [25], we determined the optimum growth conditions for each bacterium in our experiments. *Streptococcus gordonii* ATCC 10558 cells cultivated in sterilized brain heart infusion broth (BHI broth; Bacto™ Brain Heart Infusion, BD, Franklin Lakes, NJ, USA) were harvested through centrifugation, resuspended in fresh media and adjusted to a bacterial concentration of approximately 4.8 × 10^4^ colony forming units (CFU)/mL, corresponding to an optical density of 0.03 at 600 nm. The silver-coated and uncoated discs were sterilized by ethylene oxide and incubated with 1 mL of the bacterial suspension under aerobic conditions at 37 °C in 12-well plates, and bacterial growth was assessed after 24 h. Cultured *Fusobacterium nucleatum* ATCC 25586 cells in sterilized BHI broth under anaerobic conditions (80% N_2_, 10% H_2_, 10% CO_2_) were harvested and washed with fresh medium. Then, the cells were adjusted to an optical density of 0.064 at 600 nm, corresponding to a bacterial concentration of approximately 2 × 10^8^ CFU/mL. The sterilized specimens were inoculated with 1 mL of the bacterial suspension and 3 mL of fresh media in 12-well plates and incubated in an anaerobic chamber at 37 °C for 72 h. *Porphyromonas gingivalis* ATCC 33277 cells cultivated in sterile BHI broth supplemented with 0.5 mg/mL hemin and 5 mg/mL vitamin K under anaerobic conditions were harvested, washed and adjusted to a concentration of 5.1 × 10^7^ CFU/mL; the suspension had an optical density of 0.01 at 600 nm. The sterilized specimens were inoculated with 2 mL of the bacterial suspension in 12-well plates and then incubated in an anaerobic chamber at 37 °C for 48 h.

After incubation, each specimen was gently rinsed using PBS to remove unadhered bacteria from the surface. Thereafter, the remaining adherent bacteria were stained with 1% crystal violet solution for 10 min, washed gently with PBS and destained using 400 μL of a destaining solution (a mixture of 80% ethanol and 20% acetone). The absorbance of each solution was then measured using a microplate spectrophotometer (Epoch 2, BioTek, Winooski, VT, USA) at 590 nm. The absorbance values were calculated per unit area of each disc.

### Silver solution experiment

Silver solutions were prepared with commercially available silver nanoparticles (ENB KOREA Co., LTD.) in PBS at concentrations of 0.00001, 0.0001, 0.01 and 0.1 wt%. After sterilizing 5 mL of the prepared silver solutions at 121 °C for 15 min, *S. gordonii*, *F. nucleatum* and *P. gingivalis* were cultured in the solutions. The absorbance of each bacterial suspension was measured over time.

### Cell proliferation assay

Human gingival fibroblasts and mouse preosteoblasts were cultured on the silver-coated and uncoated discs, and the cellular proliferation was evaluated using a 3-[4,5-dimethyl-thiazol-2-yl]-2,5-diphenyl-tetrazolium bromide (MTT) kit (TOX-1, Sigma-Aldrich, St. Louis, MO, USA). The specimens were sterilized by ethylene oxide and placed in 12-well plates. Human gingival fibroblasts (HGFs; PCS-201-018, ATCC, Manassas, VA, USA) cultured in fibroblast basal medium (PCS-201-030, ATCC, Manassas, VA, USA) using growth kit-low serum (PCS-201-041, ATCC, Manassas, VA, USA) were collected and seeded onto the specimens at a density of 10^5^ cells/mL. After 24 h of incubation, the medium was replaced with another freshly prepared medium containing MTT in each well, and the 12-well plates were incubated at 37 °C for 4 h. After removing the medium containing MTT in each well, an MTT solubilization solution was added, and the absorbance at 570 nm was measured using a microplate reader (Epoch 2, BioTek). The background absorbance at 690 nm was also measured and subtracted from the 570 nm measurement values.

Mouse preosteoblast cells (MC3T3-E1, ATCC, Manassas, VA, USA) cultured in α-minimal essential medium containing 10% fetal bovine serum and 1% penicillin/streptomycin were also seeded on the sterilized specimens in the 12-well plates. After 4 and 7 days of culture, MTT assays were performed as described above. The negative controls were prepared by culturing the cells without specimens and the absorbance values were expressed as a percentage of the controls.

### Statistical analysis

The Shapiro–Wilk test was used to assess the normality of variables and the Levene’s test was performed to assess the equality of variances. Since the results showed non-normal distribution of the measurement values, the comparison of the biofilm formation and cell proliferation between silver-coated and uncoated surfaces of each material was carried out using the Mann–Whitney U test. Version 26.0 of the IBM SPSS Statistics (IBM Corp., Armonk, NY, USA) software was used for all statistical analyses, and the level of significance was set at 0.05.

## Results

### Surface characterization

Analysis of the elemental components of the coated surfaces using electron dispersive X-ray spectroscopy confirmed the presence of silver on both titanium and zirconia surfaces (Table 1). The arithmetic mean heights of the surfaces of the coated specimens are listed in Table 2. The silver coating was found to significantly increase the surface roughness of both materials, and no significant difference was observed when comparing the two materials. The amount of silver released from the silver-coated titanium was 0.067 ± 0.020 ppm and that from the silver-coated zirconia was 0.110 ± 0.033 ppm.

**Table 1:** Elemental analysis using electron X-ray dispersive spectroscopy

Silver-coated titanium			Silver-coated zirconia		
Element	wt%	atomic%	Element	wt%	atomic%
C	3.88	6.52	O	25.24	64.84
O	45.33	57.17	Al	1.37	2.08
Al	46.12	34.48	Y	3.65	1.69
Ti	4.09	1.72	Zr	69.29	31.22
Ag	0.58	0.11	Ag	0.45	0.17
Total	100	100	Total	100	100

*C* carbon, *O* oxygen, *Al* aluminum, *Ti* titanium, *Ag* silver, *Y* yttrium, *Zr* zirconium

**Table 2:** Arithmetic mean heights of the silver-coated specimens

	Smooth titanium	Silver-coated titanium	Smooth zirconia	Silver-coated zirconia
Sa (μm)	0.000 ± 0.000	0.220 ± 0.022	0.001 ± 0.000	0.208 ± 0.018

Values are shown as the mean ± standard deviation

*Sa* arithmetic mean height of the surface

### Crystal violet assay

**Fig. 1:**
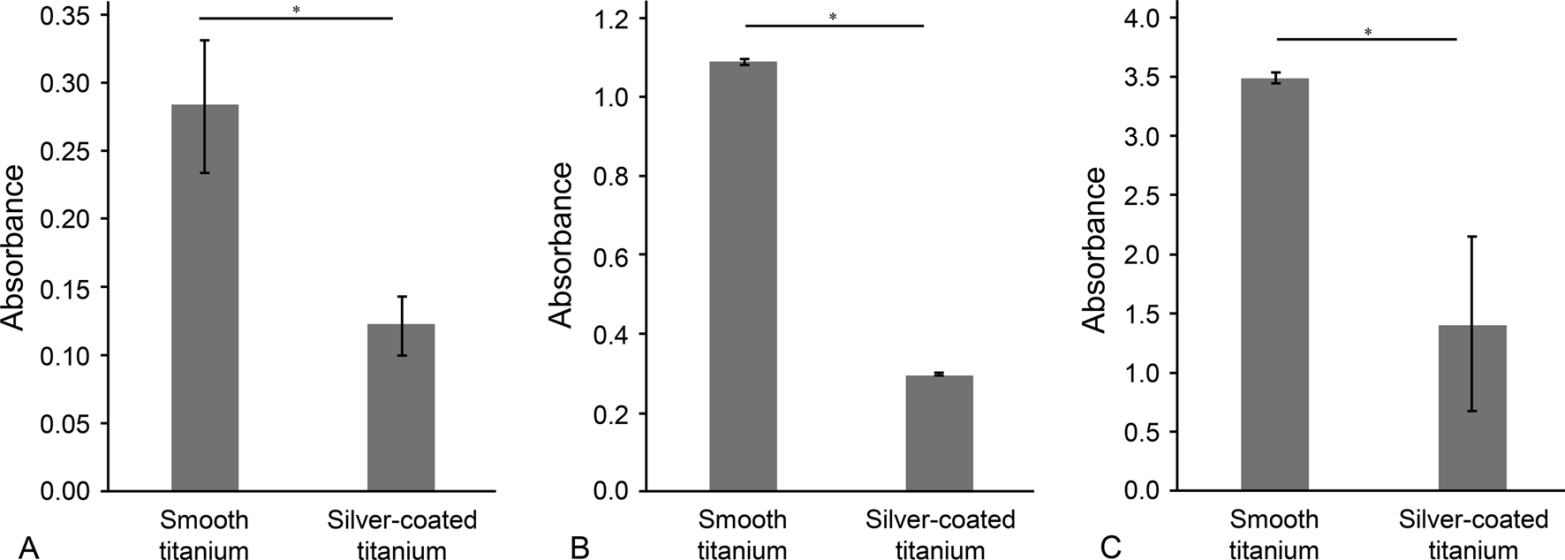
Absorbance of the crystal violet solution on titanium surfaces incubated with **A** *S. gordonii* for 24 h, **B** *F. nucleatum* for 72 h and **C** *P. gingivalis* for 48 h. The bars and intervals represent the mean absorbance values and standard deviations, respectively (*n* = 3; *p < 0.05)

**Fig. 2:**
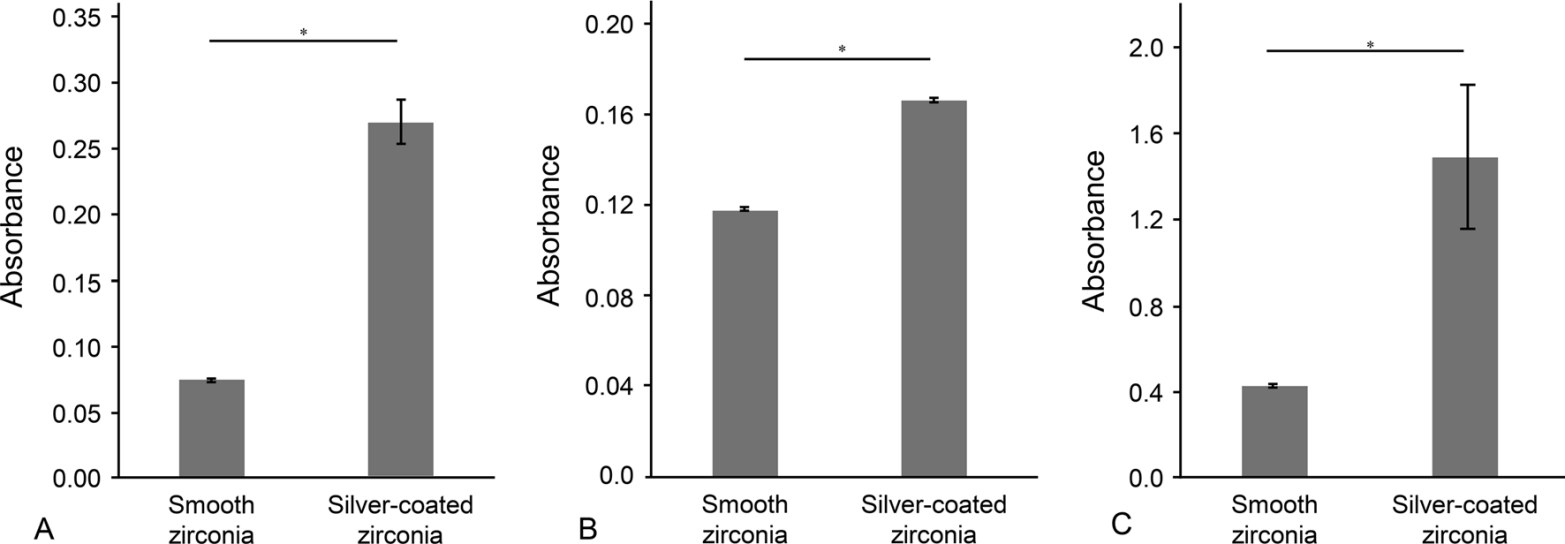
Absorbance of the crystal violet solution on zirconia surfaces incubated with **A** *S. gordonii* for 24 h, **B** *F. nucleatum* for 72 h and **C** *P. gingivalis* for 48 h. The bars and intervals represent the mean absorbance values and standard deviations, respectively (*n* = 3; *p < 0.05)

The three bacteria exhibited the same tendencies in biofilm formation. There was a significant decrease in the biofilm formation on the silver-coated titanium surface compared to the uncoated bare titanium surface and a significant increase on the silver-coated zirconia surface compared to the uncoated zirconia surface (Figs. 1 and 2).

### Silver solution experiment

**Fig. 3:**
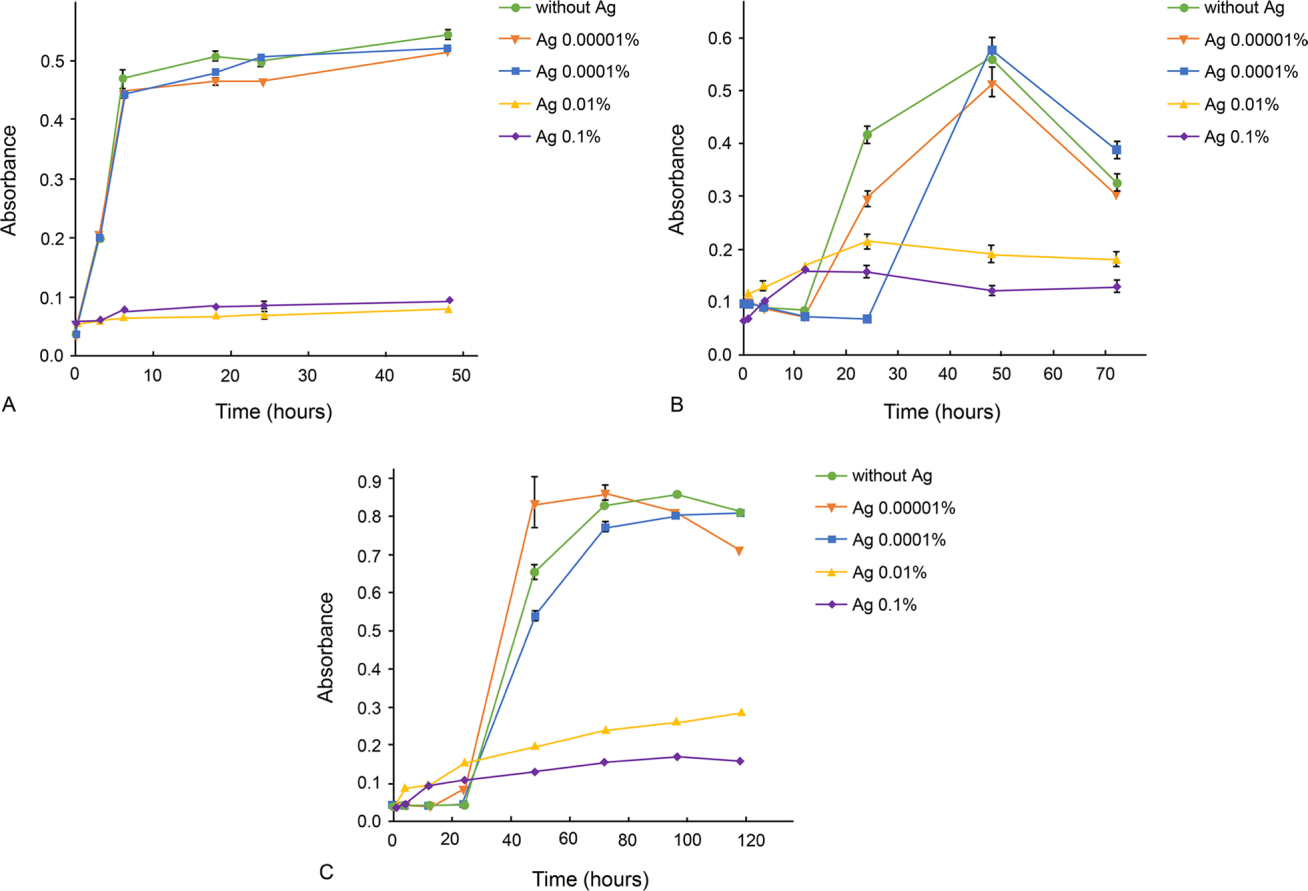
Growth curves of **A** *S. gordonii*, **B** *F. nucleatum* and **C** *P. gingivalis* cultured in 0.00001%, 0.0001%, 0.01% or 0.1% silver solution. *Ag* silver

Figure 3 shows the growth curves of *S. gordonii, F. nucleatum* and *P. gingivalis* cultured in 0.00001, 0.0001, 0.01 and 0.1 wt% silver solutions. The bacterial growth was unaffected by 0.0001 wt% silver solution, whereas concentrations above 0.01 wt% were shown to inhibit bacterial growth.

### Cell proliferation assay

**Fig. 4:**
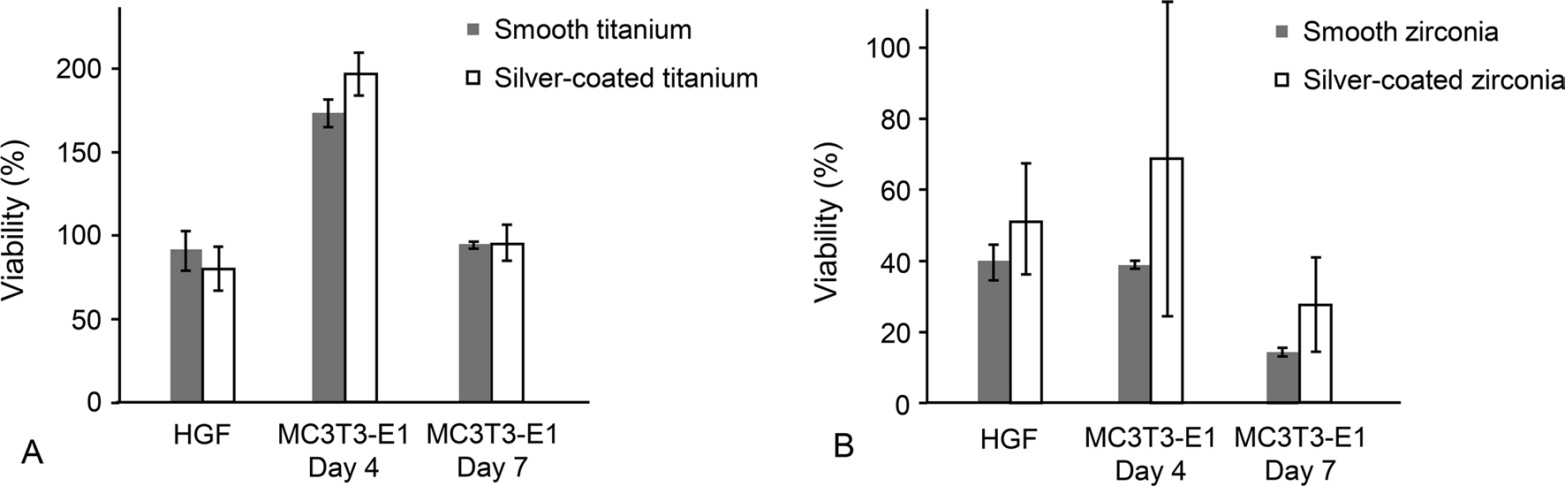
Viability of human gingival fibroblasts (HGF) for 24 h and mouse preosteoblasts (MC3T3-E1) for 4 and 7 days in MTT assay for **A** titanium and **B** zirconia specimens. The values are presented as the percentage of control. The bars and intervals represent the mean values and standard deviations, respectively. *MTT* 3-[4,5-dimethyl-thiazol-2-yl]-2,5-diphenyl-tetrazolium bromide

The absorbance measured in the MTT assay indicated the proliferation of cells cultured on each specimen (Fig. 4). For both titanium and zirconia, there was no significant difference in the cell proliferation between the silver-coated and uncoated specimens. The silver coating via aerosol deposition using a 1 wt% silver mixture on titanium and zirconia surfaces showed no cytotoxic effect on the proliferation of the HGF and MC3T3-E1 cells.

## Discussion

Since bacterial colonization on implant surfaces is closely related to implant survival rate, many studies have been performed to suppress the growth of bacteria by antibacterial application to the implant surfaces [14, 27]. In this study, we investigated silver coating as an antibacterial treatment for implant surfaces. Silver is one of the most commonly used antimicrobial agents with various clinical applications. It has been shown to have a broad-spectrum antibacterial effect, high efficacy even at low concentrations, and stability enabling straightforward application on surfaces using various techniques [14, 28–30]. Although the underlying mechanisms remain unclear, it has been widely accepted that the antibacterial properties of silver can be attributed to the silver ion, which contributes to oxidative stress, protein dysfunction and membrane damage in bacteria [15, 29]. In addition, according to recent research, silver nanoparticles themselves also have antibacterial effects by binding to the bacterial cell membrane [16].

The amount of the biofilm formation on surfaces was investigated by crystal violet staining assay. The absorbance values in crystal violet assay are directly proportional to the amount of biofilm formation on the surfaces. The results showed that the smooth titanium surface before coating was a favorable substrate for bacterial colonization, whereas the biofilm formation was significantly reduced after silver coating on the surface. For zirconia surfaces, the low absorbance values indicated the low level of biofilm formation on the surface, suggesting the low affinity of the surface for bacterial growth. After the silver coating, bacterial growth on the surface was significantly increased. The bacterial colonization on surfaces is affected by several surface properties such as surface roughness, hydrophobicity, charge and chemical composition [31–33]. Since the silver coating of the surfaces can alter the surface properties, the biofilm formation of the coated surface might be affected by the coating.

After silver nanoparticles mixed with alumina at a concentration of 1 wt% were coated onto polished surfaces via aerosol deposition, the surface roughness was significantly increased up to 0.250 μm. Surface roughness is a critical factor affecting bacterial adhesion on the surface. In particular, a surface roughness of 0.2 μm is the threshold roughness that does not affect bacterial adhesion and colonization even if the surface roughness is lower than it [34]. Therefore, an increase in surface roughness from nearly 0 μm on a smooth surface to more than 0.2 μm could cause a significant increase in bacterial adhesion.

Another surface property that could be altered after silver coating is surface charge. Since bacteria are often negatively charged by surface components, they colonize more readily on surfaces with positive to neutral charges than on those that exhibit negative charges [33]. Zirconia is a metal oxide that is covered with hydroxyl groups under aqueous conditions, and thus, its net surface charge is affected by the pH of the liquid where it is submerged [35]. Since the pH of the BHI broth was 7.4 ± 0.2 and the isoelectric point of zirconia was reported to be less than 7 [36], the net surface charge of the zirconia specimen was negative, which could repulse bacterial colonization on the surface. However, on the silver-coated zirconia surfaces, the silver nanoparticles released positively charged silver ions, which neutralized the negative surface charge of bare zirconia surfaces. The disruption of the negative surface charge after silver coating can potentially interrupt the low affinity of smooth zirconia surfaces for bacterial colonization, causing an increase in bacterial growth after silver coating on the surfaces. The coated silver also affects the surface charge of the titanium surface in a same way.

Thus, the surface roughness and surface charge after silver coating make the surfaces more favorable for bacterial growth. The bacterial colonization on the silver-coated surfaces would be affected by the bacteria-friendly surface properties as well as antibacterial effect of coated silver.

The amount of silver released from the coated specimens was approximately 0.1 ppm, and there was no significant difference between the titanium and zirconia substrates. To evaluate whether that amount of silver was effective in inhibiting bacterial growth in dispersed state, the bacteria were cultured in silver solution at the same concentration of released silver, 0.00001 wt%. Higher concentrations were also experimented to explore the effective silver concentration for antibacterial activity. The results show that the amount of silver released from the coated surface is insufficient to inhibit bacteria growth in solution, and concentrations of silver solution above 0.01% can exhibit antibacterial effect. The difference in effective concentration between the coated surface and the solution may indicate difference in the antibacterial mechanism of silver in the coated surface and solution.

The surfaces of dental implants are specifically engineered for efficient osseointegration and mucosal sealing. The incorporation of silver via aerosol deposition could interrupt the cellular response to the implant surfaces. However, the coating via aerosol deposition using a 1 wt% silver mixture had no significant effect on the fibroblasts and osteoblasts, which implied its biocompatibility. In this study, human gingival fibroblasts and mouse preosteoblasts cultured in zirconia specimens showed lower viability than controls. The results in MTT assay can be affected by the several experimental conditions such as seeding cell number, the concentration of MTT reagent and incubation time with MTT [37]. Thus, the absolute value should be analyzed with caution.

Our study was limited to the colonization of a single bacterium on the implant surfaces. As bacterial species typically form complex structures in the oral environment, the adhesion and proliferation of bacterial complexes on implant surfaces need to be further investigated. Another limitation was the differing dimensions of the titanium and zirconia discs. Directly comparing the bacterial adhesion properties between titanium and zirconia was unfeasible because these two materials were not exposed to the same experimental conditions due to their distinct disc sizes.

## Conclusions

Within the limits of the in vitro study, the results implied that the silver coating on the titanium surfaces via aerosol deposition provided antibacterial effects against oral bacteria, whereas the coating had an adverse effect on the zirconia surfaces, promoting more bacterial growth. The coating was biocompatible on both titanium and zirconia surfaces with respect to the proliferation of fibroblasts and preosteoblasts.

## Data Availability

All data generated or analyzed during this study are included in this paper.

## Abbreviations

PBS: Phosphate-buffered saline
BHI: Brain heart infusion
CFU: Colony forming units
*S. gordonii*: *Streptococcus gordonii*
*F. nucleatum*: *Fusobacterium nucleatum*
*P. gingivalis*: *Porphyromonas gingivalis*
MTT: 3-[4,5-Dimethyl-thiazol-2-yl]-2,5-diphenyl-tetrazolium bromide
HGF: Human gingival fibroblast
MC3T3-E1: Mouse preosteoblast
